# Perceptions about protein supplements among adults in Saudi Arabia (knowledge and attitude, use, health beliefs, and safety concerns)

**DOI:** 10.7717/peerj.21545

**Published:** 2026-07-29

**Authors:** Shatha Alaoufi, Raghad M. Alhomaid

**Affiliations:** 1 Department of Early Childhood, College of Education, Qassim University, Buraydah, Qassim, Saudi Arabia; 2 Department of Food Science and Human Nutrition, College of Agriculture and Food, Qassim University, Buraydah, Qassim, Saudi Arabia

**Keywords:** Protein supplements, Whey protein, Diet, Public health, Nutrition, Food Safety, Diet supplementation

## Abstract

**Background:**

Protein supplements have gained popularity among gym users in Saudi Arabia. This study aimed to collect data from adults in Saudi Arabia focusing on six components related to protein supplements: demographic characteristics of participants, health and physical activity information, knowledge and attitudes, use, health beliefs, and safety concerns.

**Method:**

This study was a cross-sectional, observational online survey targeting adults in Saudi Arabia. Participants included males and females aged 18 and older. Participants who did not complete the survey, as well as pregnant or breastfeeding women, were excluded. Ethical approval was obtained from the Deanship of Scientific Research at Qassim University. Data were analyzed by gender using SPSS version 25, with descriptive statistics and chi-square tests. A total of 358 participants completed the online questionnaire, while two participants did not complete it between March 29, 2024 and December 31, 2024.

**Result:**

Regarding knowledge and attitudes, about 68.2% of participants (including both users and non-users of protein supplements) were aware of protein supplements, with males (81.3%) showing significantly higher awareness than females (63.7%). Additionally, 64.8% of participants (both male and female) were unaware of the risks associated with protein supplements. Of all participants, 56% of males and 39.3% of females did not believe that natural protein sources are enough for muscle building, indicating a significant difference. About 127 participants (35.5%) reported using protein supplements either currently or in the past, with no significant difference between males and females. Of those who reported using protein supplements, 11.8% experienced some side effects and were significantly less knowledgeable about the amount of protein they should consume from food and how much protein is enough for their needs. The majority of protein supplement users (48.8%) did not know if protein powders could contain doping agents, with females reporting significantly higher responses than males. Regarding safety concerns, only 43.3% of protein supplement users looked for protein supplements marked with NSF Certified for Sport^®^ on the package, with no significant difference between males and females. Finally, 78% of protein supplement users expressed a need for more information on how to select the best protein supplements, with the highest need found in females and participants who exercise regularly, who showed significantly higher needs for additional guidance.

**Conclusions:**

There remains a significant need for increased education about the proper use of protein supplements to minimize potential side effects and increase understanding of their health risks and safety concerns.

## Introduction

Protein supplement use is a growing trend among adults worldwide. Common protein supplements such as creatine and protein powders are used to provide energy, to increase muscle mass, and to enhance performance and recovery (Kreider et al., 2017; Lam et al., 2019). According to 21 C.F.R. § 184.1979c (2023), whey protein involves extracting at least 25% of protein from whey using specialized techniques to eliminate unwanted materials.

A study conducted on 31 participants found a significant decreases in body mass index (BMI), waist circumference, and body fat percentage, along with an increase in muscle mass after participants consumed protein supplements for three months (20 g in 200 ml of milk/day) compared with the control group (Ambulkar et al., 2023). However, adverse effects related to dietary supplements in general are common. The same study (though it had a small sample size) revealed that three participants experienced side effects such as bloating and hyperacidity (Ambulkar et al., 2023). In the United States, it is estimated that approximately 23,000 emergency room visits were due to side effects from dietary supplements between 2004 and 2013 (Geller et al., 2015). The Saudi Food and Drug Authority (SFDA, 2020) does not guarantee the safety of dietary supplements. Wang et al. (2021) raised a health concern that branched-chain amino acids (BCAAs) could lead to type 2 diabetes, and another study indicated an association between BCAAs and non-alcoholic fatty liver disease (NAFLD; Goffredo et al., 2017). Additionally, safety concerns related to protein supplements were discussed in a study by Alaedini et al. (2021), which explained that some protein supplements were tainted with doping agents—illegal substances that could impact athletes’ future health. An Algerian study found that 31% of sport supplement users experienced side effects such as diarrhea and constipation (Chebaiki, Bekadi & Bechikh, 2020).

Previous studies have also examined the use of protein supplements. One study found that males in Riyadh, Saudi Arabia, consumed significantly more protein supplements than females (Alhakbany et al., 2022). Another study conducted in Riyadh revealed that most participants used whey protein, and 28% of the general population consumed more than two g/kg/day of protein (Alhekail et al., 2018). Moreover, a study conducted in Riyadh found that 36.3% of 502 female gym users used protein supplements, and 53.9% reported using them to build muscle mass (Alhussain et al., 2022). A recent study in Saudi Arabia showed that undergraduate students aged 18-25 spend between USD 25 and 250 each month on protein supplements, significantly impacting their finances (Almudaihim et al., 2024). In 2016, Saudi Arabia launched Vision 2030, which included the goal of increasing population participation in sports from 13% to 40% by 2030 (Saudi Vision 2030, 2025). Additionally, empowering women in sports is included as part of Saudi Arabia’s women empowerment initiatives within the Saudi annual budget (Ministry of Finance, 2023). Given these new priorities, the demand for protein supplements is expected to grow, giving adults more options and exposure to both useful and misleading information.

Although many studies have been conducted in Saudi Arabia on protein supplements, most have focused on assessing the prevalence, knowledge, attitudes, and practices related to their use. However, the present study also examined health beliefs and safety concerns, drawing on current research on protein supplements and targeting both genders.

This study provides a comprehensive overview of protein supplements focusing on six components: demographic characteristics of participants, health and physical activity information, knowledge and attitudes, use, health beliefs, and safety concerns.

The results of this study provide a comprehensive overview of the current knowledge of protein supplements and can help in the development of programs or health education campaigns aimed at increasing awareness of safe protein supplements use. 

## Methodology

### Study design and participants

This study was an observational cross-sectional online survey conducted among adults in Saudi Arabia. The survey was created using Google Forms in two languages (Arabic and English). The link was distributed to the adult population in Saudi Arabia through email and social media platforms such as WhatsApp, Twitter (X), Telegram, and Snapchat from March 29, 2024, to December 31, 2024. It should be noted that the use of social media does not represent the general population, and the results of this article cannot be generalized to the general population in Saudi Arabia.

The online questionnaire was created by the authors, the content validity of the questionnaire was reviewed by three experts in the field to ensure that all items accurately represented the concepts being measured. Experts’ feedback was consistent across several areas, including eliminating double-barreled questions, removing bias, improving answer options, and ensuring proper flow.

A pilot test was conducted to establish face validity, with 20 people to confirm that participants understood the questions as intended before the questionnaire was distributed to participants. No modifications were made, and the pilot study sample was included in the analysis.  

Internal consistency reliability was evaluated using Cronbach’s alpha. Each section of the questionnaire (health information (exercise), knowledge and use of protein supplements, health beliefs, and safety concerns related to protein supplements) showed acceptable reliability, with Cronbach’s alpha values (0.70, 0.71, 0.70, and 0.72), respectively.

Adults aged 18 and above (male or female) were included in the study.

Pregnant or breastfeeding women and participants who did not complete the survey were excluded from the study. Data was collected using a questionnaire. The sample size was calculated using the following equation (Thompson, 2012):

\begin{eqnarray*}\mathrm{where}:n= \frac{Np(1-p)}{(N-1)} \end{eqnarray*}

*n* = required sample size

N = population size

p = estimated prevalence/proportion

d = acceptable error

z(-score) = confidence level

If *N* = 21,229,785, z-score = 95%, *d* = 0.5, and *p* = 65.9%, the calculated sample size is 346. In this study, 360 participants were enrolled, and 358 completed the study.

### Questionnaire and data collection

An invitation was sent to all adults through smartphones *via* a link. To participate in the study, individuals must return a signed consent form *via* their smartphones. The self-administration questionnaire was used. The questionnaire included six parts: demographic characteristics, health information, knowledge of protein supplements, usage of protein supplements, health beliefs, and safety concerns related to protein supplements. The online questionnaire was designed by the authors and validated by other scientists in the field. The questionnaire was tested for accuracy and reliability with 20 people before being distributed to study participants.

The questionnaire was divided into the following sections:

Demographic information: gender; age; education; marital status; nationality.

Health information: chronic diseases; exercise habits.

Knowledge of protein supplements: know about protein supplements; believe that consuming protein from natural resources is enough to build muscle mass; believe that protein supplements can be consumed as a substitute for meals; believe that only men can use protein supplements; believe that athletes should take protein supplements; believe that protein supplements are good for your health; know the risks associated with protein supplements; believe that protein supplements are suitable for those with kidney and liver problems.

Health beliefs: believe protein powders could be tainted with heavy metals such as lead, arsenic, cadmium, and mercury; believe protein powders could contain doping agents, such as androgenic anabolic steroids (AAS); believe a high protein diet could affect the kidneys (hyperfiltration); believe protein powders could cause stomach aches and bloating; believe protein supplements could affect the gut microbiota.

Safety concerns: believe that all protein supplements are safe; read the package to check the content of the protein supplements; need more information on how to choose the best protein supplements; look for protein supplements that are tested by a third party; look for protein supplements that have NSF Certified for Sport^®^ on the package; check if the protein supplements are tested for banned substances; feel that the information provided by the SFDA (website or app/X) regarding protein supplements is helpful; feel that the information provided by the ministry of health regarding protein supplements is helpful.

### Statistical analysis

IBM SPSS Statistics for Mac, version 25.0 (IBM Corp., Armonk, NY, USA), was used for statistical analysis of data and to characterize the participants in the study through descriptive statistics. Data is presented as percentages in frequency tables for each part of the questionnaire to identify participants’ demographic characteristics, health information, knowledge of protein supplements, uses of protein supplements, health beliefs, and safety concerns. A chi-square test was conducted to determine the association between gender and each part of the questionnaire. *p*, representing the Pearson Chi-Square value, was considered significant at *p* ≤ 0.05. The number in brackets is the effect size (Cramer’s V).  Additional factors such as age, education, geographic region, and exercise habits were assessed by a chi-square test in relation to some questions in each component of the study.

### Ethics statement

This study was done in accordance with the Declaration of Helsinki. Ethical approval for this study was obtained from the Deanship of Scientific Research at Qassim University on 24-97-04. All participants provided their electronic informed consent to participate in the study.

## Results

### Demographic characteristics of participants

Of the 360 initial participants, 358 completed the questionnaire. Most were female (74.6%), with 25.4% male. About half were aged 18–25 (52.8%), followed by 26–5 (22.3%), 36–45 (12.6%), and over 45 (12.3%). Most held a bachelor’s degree (66.8%), 19.8% had a high school diploma, and 13.4% had postgraduate degrees. Most were single (65.9%), and 31.8% were married. Over 92% were Saudi, with Riyadh, Makkah, and Qassim comprising 20.7%, 24.9%, and 54.4%, respectively (Table 1).

**Table 1:** Demographic characteristics.

	**All**	
	*n*	**%**
Gender		
Female	267	74.6
Male	91	25.4
Age		
25–18 years	189	52.8
35–26 years	80	22.3
45–36 years	45	12.6
Over 45 years	44	12.3
Education		
High School	71	19.8
Bachelor	239	66.8
Post Graduate	48	13.4
Social status		
Widow	2	0.6
Single	236	65.9
Married	114	31.8
Divorced	6	1.7
Nationality		
Saudi	331	92.5
Non-Saudi	27	7.5
Region		
Riyadh	74	20.7
Makkah	89	24.9
Qassim	195	54.4

**Notes.**

Data are presented as Number (*n*) and Percentage (%).

Data from 358 participants who completed the questionnaire are presented; two participants did not complete it.

### Health and physical activity information

Table 2 shows that most participants were free of chronic diseases, with no significant gender differences. Regular physical activity was more common among males, who also reported exercising more frequently and at higher self-rated fitness levels compared with females. In addition, exercise settings differed significantly by gender: males predominantly exercised at gyms, whereas females were more likely to exercise at home.

**Table 2:** Health and physical activity information.

	**All**		**Female**		**Male**		*p* **(** **Cramer’s V)**
	*n*	**%**	*n*	**%**	*n*	**%**	
Do you suffer from chronic diseases?							
No	324	90.5	239	89.5	85	93.4	0.274 (0.058)
Yes	34	9.5	28	10.5	6	6.6	
Do you exercise regularly?							<0.0001 (0.263)
No	234	65.4	194	72.7	40	44	
Yes	124	34.6	73	27.3	51	56	
If yes, how many times do you exercise regularly?							<0.0001 (0.312)
1–3 days a week	39	31.4	30	41.1	9	17.6	
3–5 days a week	61	49.2	32	43.8	29	56.9	
5–7 days a week	24	19.4	11	15.1	13	25.5	
	124	100.0	73	100	51	100	
If you exercise, what is your training level?							<0.0001 (0.344)
Beginner	15	12.1	15	20.5	0	0.0	
Intermediate	66	53.2	34	46.6	32	62.7	
Advanced	39	31.5	23	31.5	16	31.4	
Expert	4	3.2	1	1.4	3	5.9	
	124	100.0	73	100.0	51	100.0	
If you exercise, where do you exercise?							<0.0001 (0.482)
At the gym	83	60.1	43	46.8	40	87	
At your house	44	31.9	42	45.6	2	4.3	
Out side	11	8	7	7.6	4	8.7	
	138	100	92	100	46	100	

**Notes.**

Data are presented as Number (*n*) and Percentage (%). The chi-squared test was used to compare all variables by gender. *p*, representing the Pearson Chi-Square value, was considered significant at *p* ≤0.05. The number in brackets is the effect size (Cramer’s V).

Data from 358 participants who completed the questionnaire are presented; two participants did not complete it.

### Knowledge and attitude of protein supplements (protein users and non-protein users)

**Figure 1:**
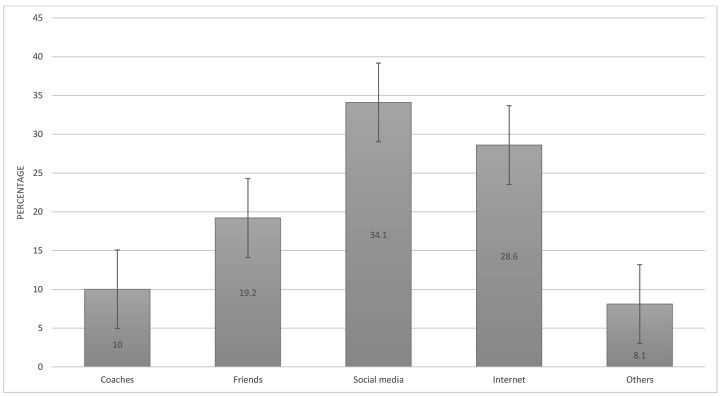
Source of knowledge about protein supplements. The main source of knowledge about protein supplements of 358 (protein users and non-users). The main sources of knowledge about protein supplements in this survey were social media, internet, friends, coaches, and others.

Most participants, especially males, know about protein supplements, mainly from social media, online platforms, friends, and coaches, as shown in Fig. 1. Significant gender differences were observed in perceptions regarding the necessity of protein supplements for muscle development, with males more likely to believe that natural dietary sources alone are insufficient. Most participants did not consider protein supplements to be meal replacements, while opinions regarding their use among athletes and their overall health benefits were mixed. Females were more likely to support supplement use among athletes, whereas males more often perceived protein supplements as healthy. Notably, knowledge regarding potential health risks was limited across both genders, with many participants unaware of possible adverse effects. In addition, most participants recognized that protein supplements may not be appropriate for individuals with kidney or liver disease (Table 3).

**Table 3:** Knowledge and attitude of protein supplements.

	**All**		**Female**		**Male**		*p* **(Cramer’s V)**
	*n*	**%**	*n*	**%**	*n*	**%**	
Do you know about protein supplements?							0.002 (0.165)
No	114	31.8	97	36.3	17	18.7	
Yes	244	68.2	170	63.7	74	81.3	
Do you believe that consuming protein from natural resource is enough to build muscle mass?							0.001 (0.193)
No	156	43.5	105	39.3	51	56	
I don’t know	65	18.2	59	22.1	6	6.6	
Yes	137	38.3	103	38.6	34	37.4	
		100		100		100	
Do you believe that protein supplements can be consumed as a substitute for meals?							0.256 (0.087)
No	263	73.5	200	74.9	63	69.2	
I don’t know	25	7	20	7.5	5	5.5	
Yes	70	19.5	47	17.6	23	25.3	
Do you believe that only men can use protein supplements than women?							0.832 (0.32)
No	237	66.2	179	67	58	63.7	
I don’t know	53	14.8	39	14.6	14	15.4	
Yes	68	19	49	18.4	19	20.9	
Do you believe that athletes should take protein supplements?							<0.0001 (0.225)
No	134	37.4	83	31.1	51	56	
I don’t know	39	10.9	33	12.4	6	6.6	
Yes	185	51.7	151	56.5	34	37.4	
Do you believe that protein supplements are good for your health?							0.055 (0.127)
No	87	24.3	73	27.3	14	15.4	
I don’t know	111	31	82	30.7	29	31.9	
Yes	160	44.7	112	42	48	52.7	
Do you know the risk associated with protein supplements?							0.616 (0.026)
No	232	64.8	175	65.5	57	62.6	
Yes	126	35.2	92	34.5	34	37.4	
Do you believe that protein supplements are suitable for those with kidney and liver problems?							0.391 (0.072)
No	214	59.8	164	61.4	50	54.9	
I don’t know	115	32.1	84	31.5	31	34.1	
Yes	29	8.1	19	7.1	10	11	

**Notes.**

Data are presented as Number (*n*) and Percentage (%). The chi-squared test was used to compare all variables by gender. *p*, representing the Pearson Chi-Square value, was considered significant at *p* ≤0.05. The number in brackets is the effect size (Cramer’s V).

Data from 358 participants who completed the questionnaire are presented; two participants did not complete it.

#### Additional factors

Additional factors assessed knowledge about protein supplements. The youngest age group (49.6%) reported strong beliefs that only men use supplements, athletes should take them, and they are suitable for those with kidney and liver issues, with 70.6%, 53%, and 58.6%, respectively (Data shown in Supplementary Files).

Moreover, individuals with a bachelor’s degree (67.2%) showed higher responses than those with other education levels. Participants from Qassim (52.9%) reported the highest responses and had strong beliefs about athletes taking supplements and awareness of their risks (57.3%, 59.5%; Data shown in Supplementary Files).

The study found that among regular exercisers, 43.9% had lower responses and beliefs about protein supplements; 43.8% believed natural sources were sufficient; 42.9% thought supplements could replace meals; and 29.2% supported supplement use for athletes. About 46.3% of exercisers saw benefits, while 44.4% expressed concern, indicating a knowledge gap among them (Data shown in Supplementary Files).

### Uses of protein supplements (protein users only)

**Figure 2:**
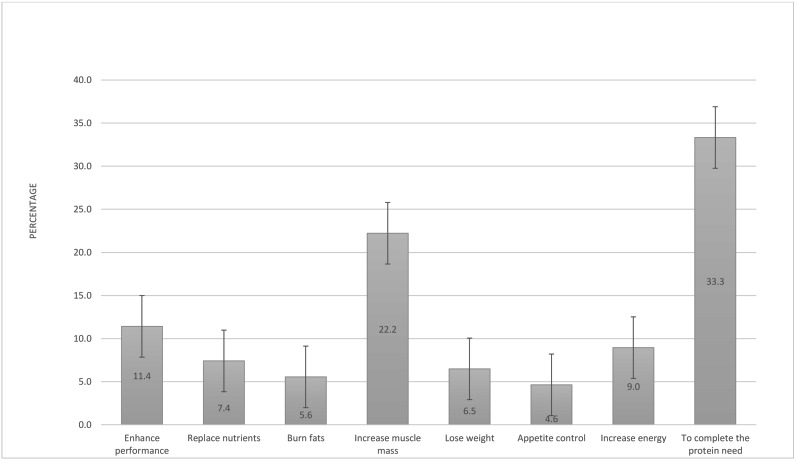
Reasons for taking protein supplements. The main reasons for taking protein supplements among participants were to meet protein needs, increase muscle mass, and enhance performance.

Table 4 shows protein supplement use, with 127 participants (35.5%) reported current or past use. The primary motivations for protein supplement use were achieving desired protein intake and increasing muscle mass, while performance enhancement was a less frequently reported reason, as in Fig. 2.

#### Additional factors

Most participants (93.7%) who used protein supplements knew about them. The youngest group (44.1%) had the highest usage, significantly higher than the other age groups: 26–35y (37%), 36–45y (15%), and over 45y (3.9%). Participants with a bachelor’s degree (61.4%) reported higher usage than those with other education levels. Those from the Qassim region (50.4%) and regular exercisers (65.4%) showed higher supplement use (Data shown in Supplementary Files).

Among protein supplement users, intake patterns were largely irregular, with only a minority reporting consistent daily use. Although most participants believed they knew their daily protein requirements, accurate knowledge of recommended intake levels was limited, with males demonstrating greater responses than females. Significant gender differences were also observed in supplement-use patterns and daily water intake. Reported adverse effects were low, though some participants remained uncertain whether they had experienced side effects.

Among participants without side effects, 80.4% knew their daily protein intake from food, 80% knew the protein amount they considered sufficient, and 79.6% knew their total daily protein needs from supplements or food for deficit build or muscle maintenance, compared to those who experienced side effects. (Data shown in Supplementary Files).

**Table 4:** Uses of protein supplements.

	**All**		**Female**		**Male**		*p* **(Cramer’s V)**
	*n*	**%**	*n*	**%**	*n*	**%**	
Are you currently taking protein supplements or have you taken before?							<0.0001 (0.224)
No	231	64.5	189	70.8	42	46.2	
Yes	127	35.5	78	29.2	49	53.8	
How long have you been using protein supplements?							0.131 (0.259)
3-4 months	11	8.7	5	6.4	6	12.2	
5-6 months	4	3.1	3	3.9	1	2.1	
More than 6 months	30	23.6	13	16.7	17	34.7	
Less than a month	6	4.7	5	6.4	1	2	
Irregular	62	48.8	42	53.8	20	40.8	
1–2 months	14	11.1	10	12.8	4	8.2	
		100		100		100	
Do you know the amount of protein you should consume from food in a day?							0.005 (0.250)
No	30	23.6	25	32.1	5	10.2	
Yes	97	76.4	53	67.9	44	89.8	
How much protein do you think is enough for your daily need?							0.016 (0.285)
0.8-1g/kg of body weight	45	35.4	29	37.1	16	32.7	
2-2.5g/kg of body weight	53	41.8	25	32.1	28	57.1	
More than 5 g/kg of body weight	6	4.7	5	6.4	1	2	
I don’t Know	23	18.1	19	24.4	4	8.2	
		100		100		100	
How many times do you use protein supplements?							0.006 (0/313)
Only When I have a competition	5	3.9	2	2.5	3	6.1	
Workout days	47	37	24	30.8	23	46.9	
Not consistent	58	45.7	45	57.7	13	26.6	
Everyday	17	13.4	7	9	10	20.4	
		100		100		100	
How much water do you drink daily?							0.008 (0.328)
More than 8 cups	31	24.4	15	19.2	16	32.7	
1–2 cups	11	8.7	11	14.1	0	0	
3–4 cups	27	21.3	20	25.6	7	14.3	
5–6 cups	28	22	13	16.7	15	30.6	
7–8 cups	30	23.6	19	24.4	11	22.4	
		100		100		100	
Did you experience any side effects after using protein supplements?							0.309 (0.136)
No	98	77.2	57	73.1	41	83.7	
I don’t know	14	11	11	14.1	3	6.1	
Yes	15	11.8	10	12.8	5	10.2	
		100		100		100	
Did you stop using protein supplements after experiencing side effects?							0.632 (0.116)
No	2	13.3	2	20	0	0	
For a temporary period	4	26.6	3	30	1	20	
Yes	9	60.1	5	50	4	80	
Do you know the amount of protein you need in a day, whether from protein supplements or from food, to calculate your daily need or to build or maintain muscle mass							0.024 (0.200)
No	29	22.8	23	29.5	6	12.2	
Yes	98	77.2	55	70.5	43	87.8	

**Notes.**

Data are presented as Number (*n*) and Percentage (%). The chi-squared test was used to compare all variables by gender. *p*, representing the Pearson Chi-Square value, was considered significant at *p* ≤0.05. The number in brackets is the effect size (Cramer’s V).

Data from 358 participants who completed the questionnaire are presented; two participants did not complete it.

Among participants who experienced side effects, most discontinued protein supplement use, either permanently or temporarily, with no significant gender differences observed. Responses of daily protein requirements for muscle maintenance were generally high, though males had higher responses than females.

**Figure 3:**
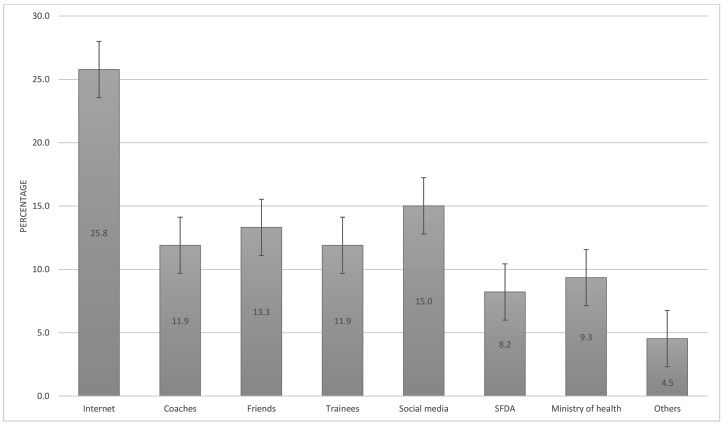
Sources of information about protein supplements. Where participants obtained information about protein supplements, with the internet being the most common source, while the SFDA and others were the least common sources.

**Figure 4:**
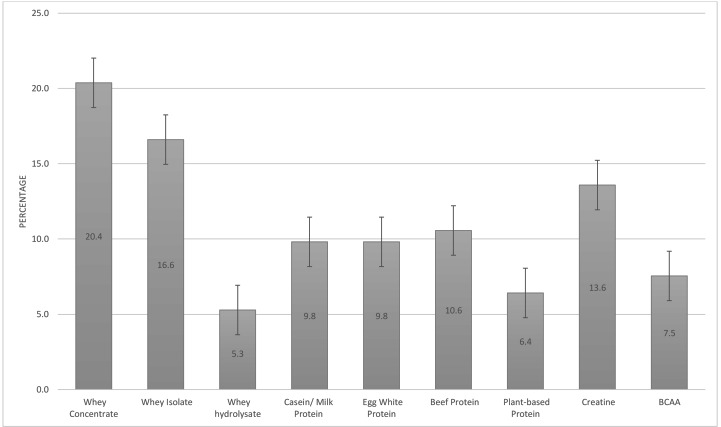
Types of protein supplements used. Different types of protein supplements used by participants. In order of popularity, whey concentrate had the highest percentage of users, followed by whey isolate, creatine, and beef protein.

Figure 3 demonstrates that online sources were the primary means through which participants obtained information about protein supplements. Figure 4 shows that whey concentrate-based products were the most commonly used supplements, followed by Whey Isolate, whereas other supplement types were used less frequently.

### Health beliefs (protein users only)

Table 5 highlights considerable uncertainty regarding the potential health risks associated with protein supplements. Many participants were unaware that protein powders may contain contaminants such as heavy metals or prohibited substances, although females were more likely than males to recognize the possibility of doping-related additives. Responses were higher about the potential adverse effects of high-protein intake on kidney function and gastrointestinal symptoms like bloating and stomach discomfort. The potential impact of protein supplements on the gut microbiota remained limited in both genders.

**Table 5:** Health beliefs.

	**All**		**Female**		**Male**		*p* **(Cramer’s V)**
	*n*	**%**	*n*	**%**	*n*	**%**	
Do you think protein powders could be tainted with heavy metals such as lead, arsenic, cadmium, and mercury?							0.001 (0.342)
No	36	28.3	13	16.6	23	46.9	
I don’t know	54	42.6	36	46.2	18	36.8	
Yes	37	29.1	29	37.2	8	16.3	
		100		100		100	
Do you think protein powders may contain Doping agents, such as androgenic anabolic steroids (AAS)?							0.025 (0.241)
No	37	29.1	16	20.5	21	42.9	
I don’t know	62	48.8	42	53.8	20	40.8	
Yes	28	22.1	20	25.7	8	16.3	
		100		100		100	
Do you think high protein diet could affect the kidneys (hyperfiltration)?							0.718 (0.072)
No	16	12.6	9	11.5	7	14.3	
I don’t know	36	28.3	24	30.8	12	24.5	
Yes	75	59.1	45	57.7	30	61.2	
		100		100		100	
Do you think protein powders could cause stomach ache and bloating?							0.093 (0.193)
No	12	9.4	5	6.4	7	14.3	
I don’t know	26	20.5	20	25.7	6	12.2	
Yes	89	70.1	53	67.9	36	73.5	
		100		100		100	
Do you think protein supplements could affect the gut microbiota?							0.247 (0/148)
No	13	10.2	7	9	6	12.2	
I don’t know	69	54.3	39	50	30	61.2	
Yes	45	35.5	32	41	13	26.6	
		100		100		100	

**Notes.**

Data are presented as Number (*n*) and Percentage (%). The chi-squared test was used to compare all variables by gender. *p*, representing the Pearson Chi-Square value, was considered significant at *p* ≤0.05. The number in brackets is the effect size (Cramer’s V).

Data from 358 participants who completed the questionnaire are presented; two participants did not complete it.

### Safety concerns (protein users only)

Table 6 reveals substantial safety concerns and uncertainty regarding protein supplement use among participants. Although most users reported reading product labels, many expressed distrust in supplement safety and indicated a need for additional guidance on selecting appropriate products, particularly females. Participants’ awareness of third-party testing certifications and of NSF Certified for Sport^®^ was moderate. No significant gender differences were observed. While checking for banned substances was inconsistent. Information provided by national health authorities, including the Saudi Food and Drug Authority and the Ministry of Health, was perceived as helpful by many participants, with females showing greater reliance on Ministry of Health resources.

**Table 6:** Safety concerns.

	**All**		**Female**		**Male**		*p* **(Cramer’s V)**
	*n*	**%**	*n*	**%**	*n*	**%**	
Do you believe that all protein supplements are safe to be used?							0.068 (0.206)
No	71	55.9	41	52.6	30	61.3	
I don’t know	29	22.8	23	29.5	6	12.2	
Yes	27	21.3	14	17.9	13	26.5	
		100		100		100	
Do you read the package to check the content of the protein supplements?							0.814 (0.021)
No	22	17.3	14	17.9	8	16.3	
Yes	105	82.7	64	82.1	41	83.7	
							
Do you feel that you need more information on how to choose the best protein supplements?							0.03 (0.235)
No	21	16.5	8	10.3	13	26.5	
I don’t know	7	5.5	6	7.7	1	2.1	
Yes	99	78	64	82	35	71.4	
		100		100		100	
Do you look for protein supplements that are tested by a third party?							0.175 (0.166)
No	39	30.7	23	29.5	16	32.6	
I don’t know	20	15.7	16	20.5	4	8.2	
Yes	68	53.6	39	50	29	59.2	
		100		100		100	
Do you look for protein supplements that have NSF Certified for Sports on the package?							0.25 (0.148)
No	34	26.8	17	21.8	17	34.7	
I don’t know	38	29.9	26	33.3	12	24.5	
Yes	55	43.3	35	44.9	20	40.8	
		100		100		100	
Do you check if the protein supplements are tested for banned substances?							0.018 (0.251)
No	51	40.2	24	30.8	27	55.1	
I don’t know	26	20.4	10	25.6	6	12.2	
Yes	50	39.4	34	43.6	16	32.7	
		100		100		100	
Do you feel that the information provided by the SFDA (website or app/X) regarding protein supplements is helpful?							0.347 (0.129)
No	17	13.4	8	10.2	9	18.4	
I don’t know	56	44.1	34	43.6	22	44.9	
Yes	54	42.5	36	46.2	18	36.7	
		100		100		100	
Do you feel that the information provided by the Ministry of Health regarding protein supplements is helpful?							0.046 (0.220)
No	13	10.2	4	5.1	9	18.3	
I don’t know	55	43.3	34	43.6	21	42.9	
Yes	59	46.5	40	51.3	19	38.8	
		100		100		100	

**Notes.**

Data are presented as Number (*n*) and Percentage (%). The chi-squared test was used to compare all variables by gender. *p*, representing the Pearson Chi-Square value, was considered significant at *p* ≤0.05. The number in brackets is the effect size (Cramer’s V).

Data from 358 participants who completed the questionnaire are presented; two participants did not complete it.

#### Additional factors

Among regular exercisers, 64.6% reported needing more info on choosing protein supplements. Most look for NSF Certified for Sports^®^ (70.9%) and check for banned substances (72%; Data shown in Supplementary Files). Participants from Qassim found SFDA and Ministry of Health info on protein supplements most helpful (72.2% and 71.2%), higher than other regions. (Data shown in Supplementary Files).

## Discussion

In this study, more males exercised regularly 3–5 days a week than females. This result is not surprising and aligns with data from The General Authority for Statistics (GASTAT) in Saudi Arabia, which shows a significant difference between males (54.8%) and females (38.3%) in exercising for at least 30 min weekly (General Authority of Statistics (GASTAT), 2022). Alhakbany et al. (2022) found that more males attend gyms than females.

In the present study, there was a significant gender difference in exercise location, with most males and fewer than half of females exercising at the gym. Alharbi et al. (2024) reported social restrictions, high gym costs, inactive public transport, time constraints, hot weather, and roads outside as barriers for Saudi women. In addition, overprotection and common beliefs also serve as barriers (Aljehani et al., 2022). Similarly, Almaqhawi (2021) identified barriers like cost, limited experience, absence of personal trainers, culture environmental factors, and transportation issues. Along with these barriers, Radhakrishnan et al. (2020) added fear of becoming muscular, overprotection, and the widespread belief that sports are only for men.

Significantly more males than females in this study knew about protein supplements, likely because they exercise more frequently than females and may hear about them from trainers or coaches at the gym. The youngest age group, especially those with a bachelor’s degree, exhibited significantly higher responses to protein supplements than older participants or those at other educational levels. This exceeds Almudaihim et al. (2024), who reported only 17% of that age group found protein supplements are popular. Younger respondents also believed they are suitable for people with kidney and liver problems, and thought only men can use them. These findings suggest the potential need for targeted educational programs about protein supplements and their associated risks.

Although most males and females in the present study knew about protein supplements, their main sources of information were social media, followed by the internet, friends, and coaches. A Saudi study found coaches as the primary source (Alhakbany et al., 2022), while Almudaihim et al. (2024) showed that the internet was the main source, followed by social media, doctors, pharmacists, and gym trainers. Social media, friends, and the internet are unreliable sources of information. Alhakbany et al. (2022) social media influenced, some athletes to use protein supplements. Instead, gym users should seek information from health professionals to avoid being misled (Saleh & Julien, 2022).

Most participants especially male, believed that consuming protein from natural resources alone is not enough to build muscle. However, almost half of those who exercised regularly thought that natural protein resources were sufficient for muscle growth. A cohort study by Alexandrov et al. (2018) found a link between protein intake from animal sources and increased muscle mass and creatine excretion (urinary creatine excretion is an indicator of muscle gain); therefore, protein could help preserve and build muscle.

Also, most participants who exercise regularly believed that protein supplements could replace meals. However, using protein powder instead of natural foods like (*e.g.*, meat, fish, or chicken) is not recommended (Antonio et al., 2024). A balanced diet with protein in daily meals is preferable.

The youngest group of participants (males and females) and those from the Qassim region had the highest rates of belief that athletes should take protein supplements among all age groups and geographic regions. Vieira et al. (2022) and Pasiakos, McLellan & Lieberman (2015) indicated that protein supplementation combined with resistance exercise can increase muscle mass. Ambulkar et al. (2023) found a significant difference between supplement users and non-users with increases in energy, aerobic performance, muscle mass, and a decreases in body mass index (BMI), waist circumference, and body fat. Only 44.4% of regular exercisers in this study expressed concerns about the risks of protein supplements. Chebaiki, Bekadi & Bechikh (2020) reported that 61% of athletes were unaware that supplements can negatively impact health and believed there were no risks. This underscores the need to educate on both benefits and risks of protein supplements.

The 35.5% prevalence in this study is close to the 39% reported in Riyadh by Alhekail et al. (2018), among 185 participants. This rate is lower than other Riyadh and Algeria studies where. In Alhakbany et al. (2022) found about 50% of participants used protein supplements, with males (68.7%) consuming significantly more protein supplements than females (35.6%). In Seville, Oliver, León & Guerra-Hernández (2011) reported 28% usage, higher in males (42.7%) than females (3.2%). In Algeria, Chebaiki, Bekadi & Bechikh (2020) reported 100% of participants consumed at least one protein supplement. This likely reflects differences between the groups, as all participants in Chebaiki, Bekadi & Bechikh (2020) were recreational or professional athletes.

In this study, the main reason participants took protein supplements was to meet their protein needs, followed by increasing muscle mass and enhancing performance. This differs from the primary reason reported by Chebaiki, Bekadi & Bechikh (2020), where the main reason was to increase muscle mass (59%) and then improve performance (25%). In addition, males were significantly more likely than females to report knowing the amount of protein they consume from food in a day and how to calculate the amount of protein needed. This indicates that some women in Saudi Arabia still need to improve their understanding of the protein amounts they need from food or supplements.

There is not a single correct or incorrect amount of daily protein intake. In 2007, the WHO/FAO suggested an average recommended intake of 0.83 g per kg of body weight per day for healthy adults, with no upper limit was determined. Consuming twice the recommended amount of protein poses no risk, but intake at 3–4 times the recommendation may not be risk-free (European Commission, 2021). However, the amount of protein needed varies based on individual needs and goals.

Nearly one-third of male participants in this study reported consuming more than 8 cups of water per day compared with less than a quarter of female participants. The European Food Safety Association (EFSA) recommends about 2.5 L/day of water for men and 2.0 L/day for women (EFSA, 2010). A study of 358 healthy participants found that water intake was associated with significant decreases in BMI, body fat mass, and waist circumference in females. This indicates that water intake affects body weight and body composition (Laja García et al., 2019).

Only 11.8% of participants in this study experienced side effects from protein supplements, similar to Millán-Jiménez et al. (2023) study reporting 18.51%. However, in the present study, those who experienced side effects were significantly less aware of how much protein they should consume from food daily, the total recommended amount of protein per day, and the daily amount of protein needed to build or maintain muscle mass. This suggests a need for more education on the proper use of protein supplements to reduce side effects.

In this study, participants were asked to identify the different types of protein supplements they used. The majority used whey concentrate, followed by whey isolate, creatine, and beef protein. Alhekail et al. (2018) found high whey protein usage among medical students (91%) and gym users (85%).

Participants in the present study mostly obtained information about protein supplements from the internet, followed by social media. Jawadi et al. (2017) reported that 38% of their participants got their information from online sources, and 35% from coaches. The reliability of sources like the internet or social media are an ongoing issue. Klein & Schweikart (2022) stated that influencers play a major role in marketing dietary supplements. While customers trust influencers, they can also spread false information and influence behavior.

Heavy metals such as mercury (Hg), cadmium (Cd), arsenic (As), chromium (Cr), nickel (Ni), zinc (Zn), copper (Cu), and lead (Pb) can enter our bodies through various sources. Soil and water may become contaminated with these metals due to human activities like mining, metallurgy, pesticide use, wastewater, fertilizers, and sewage (Schmitz et al., 2019). These toxic metals then accumulate in the human body *via* the food chain, potentially causing health problems affecting the heart, brain, and bones, and increasing the risk of cancer, kidney failure, and cognitive impairments (Mititelu et al., 2025).

Most participants in the present study were unsure if protein powders could be tainted with heavy metals. A study in India tested 36 protein supplements, including pure plant-based protein and pure whey-based protein for heavy metals and aflatoxins. Arsenic was detected in five (13.9%), cadmium in 10 (27.8%), lead in 27 (75%), and copper in 34 (94.4%). The study also found five protein products contained aflatoxins above 10 μg/kg (Philips et al., 2024). Bandara, Towle & Monnot (2020) tested 15 protein powders, finding higher heavy metal levels in plant-based protein detectable amounts of As, Cd, Hg, and Pb. The Clean Label Project tested 160 protein powders, with 47% exceeding safety limits, especially in plant-based proteins, notably with cadmium. Moreover, chocolate protein powders had more lead contamination than vanilla (The Clean Label Project, 2024).

Many participants in the present study responded that they did not know whether protein powders could contain doping agents, such as androgenic anabolic steroids (AAS). The World Anti-Doping Agency (WADA) is dedicated to combating doping and promoting doping-free sports (World Anti-Dopin Agency (WADA), 2025). Anabolic–androgenic steroids (AAS) are among the substances prohibited by WADA’s list (World Anti-Doping Agency  (WADA), 2024). A study in Riyadh found that 82% of AAS users had inadequate knowledge about the substance (Al Bishi & Afify, 2017). Also, a review of 3,132 dietary supplements found that about 875 (14 out of 50 articles) supplements contained undeclared substances, with 248 (28.34%) of these containing prohibited substances listed by WADA (Kozhuharov, Ivanov & Ivanova, 2022).

Some participants believed that a high-protein diet could affect the kidneys (hyperfiltration), and the majority responded that protein powders could cause stomach aches and bloating. A review found that excessive whey protein mainly affects the kidneys and liver (Vasconcelos, Bachur & Arag ao, 2021). Many protein powder users report stomach pain. Alhakbany et al. (2022) reported 68.5% experienced side effects like diarrhea, constipation, indigestion, nausea, stomach pain, and lack of appetite. However, high-quality protein powders can improve intestinal barrier function and regulate gut microbial imbalance, especially in patients with inflammatory bowel disease (IBD) (Li & Wang, 2024). A study of 60 participants found no adverse effects, except in three individuals who reported bloating and hyperacidity (Ambulkar et al., 2023).

More than half of the participants in the present study did not know whether protein supplements could affect the gut microbiota. Moreno-Pérez et al. (2018) investigated the impact of whey isolate and beef hydrolysate on 12 runners over 10 weeks, noting a decrease in *Bifidobacterium*. This beneficial bacterium is typically found in the intestine during early life but can decrease with age due to factors like obesity, cancer, and allergies (Arboleya et al., 2016; Voreades, Kozil & Weir, 2014). Moreno-Pérez et al. (2018) also observed an increase in the *Bacteroidetes phylum,* indicating long-term use of protein supplements might have negative effects on gut health. Conversely, an *in vitro* study examined fecal samples from healthy and obese participants after consuming various whey proteins and found an increase in *Bifidobacterium*, suggesting that whey protein might enhance intestinal health (Sánchez-Moya et al., 2017). More research is necessary to clarify the connection between protein supplements and the gut microbiota.

Contamination of protein supplements with illegal substances such as anabolic agents, stimulants, steroids, and pro-hormones was detected in some supplements in the US market. Multiple studies also highlighted concerns like mislabelling, proprietary blends, and severe side effects, (Jagim et al., 2023). In this study, some users expressed concerns about the safety of protein supplements, and half of the participants did not believe that all protein supplements are about safe to use.

Only about one-third of study participants checked the protein supplements were tested for banned substances, with higher rates reported among regular exercisers. A previous study found that 70% of 259 study participants checked if their supplements were free from banned substances (Gabriels & Lambert, 2013).

Only 43.3% of participants in the current study looked for protein supplements bearing the NSF Certified for Sport^®^ label, with higher percentages among those who exercised regularly. NSF tests supplements for prohibited substances listed by WADA to reduce the risk of exposure, and major sports organizations recommend using NSF Certified for Sport^®^ products (NSF, 2025). Additionally, this study found that over half of the participants chose third-party tested protein supplements. A Dutch study showed that 71% of Olympic athletes are aware of the Dutch third-party testing system (NZVT) when purchasing nutritional supplements, and 87.8% believe consuming mislabelled supplements is unacceptable (Schmitz et al., 2018).

In this study, participants used protein supplements from NSF Certified Sports^®^ brands and from non-certified brands (data not shown). Jagim et al. (2023) provided some safe supplement tips, such as seeking supplements with third-party verification.

Less than half of the participants felt that the information provided by the SFDA (*via* website or app/X) regarding protein supplements was helpful, with a higher percentage of participants from the Qassim region considering it useful compared to those from other regions. The SFDA offers guidelines for complementary foods for athletes, including types, goals, doses, timing of consumption, natural alternatives, and potential side effects (SFDA, 2023). Almost half of the participants valued the information from the Saudi Arabia Ministry of Health about protein supplements, with the highest appreciation among Qassim region residents. The Ministry of Health provides information about types of anabolic steroids, their side effects, and guidelines for supplement use (Ministry of Health, 2019). Despite these findings, several limitations should be considered when interpreting the results, such as an overrepresentation of females (74%), as they were more responsive than males. There is also a potential for self-report bias, such as recall bias or misinterpretation of questions. The cross-sectional design prevents measurement of behavioral changes over time. Additionally, online distribution limits the generalizability of the findings to the broader Saudi Arabian population. The regional imbalance, with high representation from Qassim due to the study being conducted there, is another limitation. Future research should include experimental or longitudinal studies to assess the impact of protein supplements on health. Furthermore, a laboratory analysis of the contamination of protein supplement available in Saudi Arabia is recommended.

## Conclusion

This study is among the first studies to include demographic characteristics, health and physical activity information, knowledge and attitudes, use, health beliefs, and safety concerns regarding protein supplements, all in one study. Health risks and safety concerns related to protein supplements are important components that need to be addressed in targeted programs aimed at increasing awareness about protein supplements, especially for females or young adults in Saudi Arabia.

## Supplemental Information

10.7717/peerj.21545/supp-1 Supplemental Information 1 Raw data.

## Additional Information and Declarations

### Data Availability

The following information was supplied regarding data availability: The raw data is available in the Supplemental File.
